# Resveratrol Inhibits Proliferation and Differentiation of Porcine Preadipocytes by a Novel LincRNA-ROFM/miR-133b/AdipoQ Pathway

**DOI:** 10.3390/foods11172690

**Published:** 2022-09-03

**Authors:** Qinyang Jiang, Sanbao Zhang, Xiaotong Gao, Yan Hu, Yu Zhang, Yujian Shen, Yuhang Jiang, Yanna Huang

**Affiliations:** College of Animal Science and Technology, Guangxi University, Nanning 530004, China

**Keywords:** *LincRNA-ROFM*, resveratrol, pig, *miR-133b*, AdipoQ, back-fat thickness

## Abstract

Resveratrol (RES) has a wide range of biological and pharmacological activities with various health benefits for humans as a food additive. In animal production, RES has been considered a potential functional feed additive for producing high-quality pork. Long noncoding RNAs (lncRNAs) have emerged as essential regulators of fat metabolism, and phytochemicals can regulate fat metabolism through lncRNA. However, it is unclear whether RES can improve back-fat thickness by regulating lncRNA. In this study, we identified a novel lncRNA, which was named a long intergenic non-protein coding RNA, a regulator of fat metabolism (*LincRNA-ROFM*), from our previous lncRNA sequencing data. *LincRNA-ROFM* can inhibit adipocyte proliferation and differentiation. In-depth analyses showed that *LincRNA-ROFM* acts as a molecular sponge for *miR-133b,* and adiponectin (AdipoQ) is a direct target of *miR-133b* in porcine preadipocytes. In addition, the expression of *LincRNA-ROFM* was positively correlated with AdipoQ. RES can promote the expression of *LincRNA-ROFM* by *PPAR*α and *C/EBP*α. Altogether, our research showed that *LincRNA-ROFM* acts as a ceRNA to sequester *miR-133b* and is upregulated by RES, leading to heightened AdipoQ expression, and thus decreased adipocyte proliferation and differentiation, which reduces back-fat thickness of pigs. Taken together, the *RES/LincRNA-ROFM/miR-133b/*AdipoQ regulatory network preliminarily explains the mechanism of action of RES in inhibiting fat deposition, which provides new insight into the downstream mechanism of RES inhibition of fat deposits by regulating the lncRNA.

## 1. Introduction

Pigs are the chief source of meat protein for people around the world [1], and pork accounts for more than one-third of human meat consumption [2]. The quality of pork being consumed is closely related to human health and is strongly influenced by fat deposition [3]. Pigs with a high proportion of subcutaneous fat produce low-quality pork. This is mainly because fat can influence the tenderness, juiciness, and palatability of the meat [4,5,6]. Rapid growth and a high lean meat percentage pig is always preferred by people [7]. Adipogenesis is controlled by a complex process that is regulated by various transcriptional events [8], including peroxisome proliferator-activated receptor γ(*PPAR-γ*) [9,10], Adiponectin (*AdipoQ*) [11], CCAAT/enhancer-binding protein (*C/EBPs*) [12], Krüppel-like transcription factors (*KLFs*) [13], zinc finger protein 423 (*Zfp423*) [14], and fibroblast growth factor 10 (*FGF10*) [15].

Long noncoding RNAs (lncRNAs, >200 nucleotides) play a key role in the growth of an organism through effects on chromatin modification, gene imprinting, and subcellular protein localization [16,17]. They participate in multiple biological processes including cell differentiation, transcriptional regulation, and maintaining cell structure [18]. In the breeding of livestock and poultry, most studies have focused on the important role of lncRNA in the regulation of phenotypes related to economic production, such as muscle development and fat metabolism [19]. Recently, a growing number of reports have demonstrated that lncRNAs can act as competing endogenous RNAs (ceRNAs) in the regulation of fat deposition [20]. ceRNAs can impair miRNA activity by acting as molecular sponges for miRNAs and thereby upregulating the miRNA target gene expression [21]. *LncRNA IMFNCR* promotes intramuscular adipocyte differentiation by sponging *miR-128-3p* and *miR-27b-3p* [22]. Adipocyte differentiation-associated long non-coding RNA (*ADNCR*) was downregulated during the differentiation of adipocytes and inhibited adipocyte differentiation by functioning as a ceRNA for *miR-204* [23]. These studies demonstrate the growing importance of lncRNAs and adipogenesis. However, our knowledge of lncRNAs related to adipocyte differentiation is limited.

With an increase in consumer demand for high-quality animal protein, it becomes imperative to improve meat quality through nutritional strategy. RES is a plant polyphenol that exists in grapes, peanuts, mulberries, pomegranates, and berries, and has been considered a potential functional food/feed additive [24]. RES has been widely reported to involve in a wide range of biological activities [25], including anti-inflammatory [26], anticancer [27], antioxidant [28,29], preventing obesity [30], and treating diabetes [31]. In animal husbandry, RES can increase the proportion of oxidative muscle fiber by the AdipoR1-AMPK-PGC-1α pathway to improve pork quality [25] and improve the growth performance, meat percentage, and meat quality of fattening goat [32]. Moreover, RES was also found to affect lipid metabolism, and regulate adipocyte proliferation, differentiation, and apoptosis [3] by PPARγ [33], PKA/AMPK/PPARα [34], and Mtor [35] pathway. Interestingly, studies have reported that RES can improve lipid metabolism and hepatic insulin resistance by downregulating *lncRNA NONMMUT008655.2* [36]. Our previous study indicated that RES can reduce subcutaneous fat deposition in pigs without affecting body weight, average daily gain and average daily feed intake [25]. Additionally, we found that the *lncRNA-MSTRG.9710* differential expressed in pig subcutaneous fat between control group and 400 mg/kg RES group by RNA-seq. However, the mechanism by which RES reduces subcutaneous fat deposition by *lncRNA-MSTRG.9710* in pigs is still not elucidated.

In this study, we aim to determine whether RES reduces subcutaneous fat deposition in pigs via *lncRNA-MSTRG.9710*. This not only further improved the regulatory network of RES involved in fat deposition pathway, but also provided a scientific basis for exploring the involvement of lncRNA in porcine fat metabolism.

## 2. Materials and Methods

The experimental protocols were approved by the Guide for the Use of Animal Subjects in Guangxi University and the Rules and Regulations of Experimental Field Management Protocols.

### 2.1. Experimental Animals and Sample Collection

All experimental animals and tissue samples were collected previously in our laboratory [25]. Briefly, twenty-four healthy crossbred castrated boars (*Duroc × Landrace × Yorkshire, DLY*) with an average initial body weight of 65.0 kg were randomly divided into four groups with six duplicates per group. The group was randomly assigned to each of four dietary treatments that consisted of a basal diet (control) and a basal diet supplemented with 200 mg, 400 mg, or 600 mg RES (provided by Wan Fang Biotechnology Co., Ltd. (Xi’an, China) with a purity of 99.8%.) per kilogram (kg) of feed. Diets were fed for 41 days. At the end of the feeding experiment, pigs were fasted for 12 h and transported to the experimental abattoir. Tissues from different parts were collected and stored in liquid nitrogen. In addition, to explore the expression differences of *lncRNA-MSTRG.9710* in subcutaneous fat of pigs at different growth stages, subcutaneous fat of healthy crossbred castrated boars DLY fed with a basic diet at 35 kg, 60 kg, and 110 kg were also collected, respectively.

### 2.2. Screening of Candidate LncRNAs

The *lncRNA-MSTRG.9710* is on the basis of our previous work in an animal science laboratory. Briefly, the subcutaneous adipose tissue of control group and 400 mg/kg RES group were performed for RNA-seq. We used FDR corrected, |log_2_FC| ≥ 1 and probability > 0.8 (multiple testing were used for eliminating false positive, probability level > 0.8 signifies that the false discovery rate (FDR) of this gene is less than 0.2 by machine-learning model) as the threshold to determine differentially expressed (DE) significantly lncRNA. In this paper, lncRNA with target gene *AdipoQ* was selected for subsequent verification.

### 2.3. RNA Extraction and Quantitative Reverse Transcription PCR (RT-qPCR)

RNAios (TaKaRa, Dalian, China) was used to extract the total RNA. After the concentration and purity of the total RNA were detected, it was reverse transcribed into cDNA by reverse transcription kit (TaKaRa, Dalian, China). Real-time PCR (RT-qPCR) was performed with cDNA as template. The PCR reaction mixture contained 5 μL of TB GreenTM Premix Ex Taq TM II (TaKaRa, Dalian, China), 0.25 μL of forward and reverse primers, respectively, 1 μg of cDNA, and 2 μL of RNase Free H_2_O in a 10 μL reaction system. The reaction procedure is as follows: pre-denaturation at 95 °C for 30 s, denaturation at 95 °C for 5 s, extension at 60 °C for 30 s, 45 cycles, extension at 65 °C for 5 s, extension at 95 °C for another 5 min. The primers used in this study were shown in Table S2 and synthesized in Sangon Biotech (Shanghai) Co., Ltd., China. All data were normalized to the 18S rRNA transcript. The mRNA abundance of each gene was calculated by 2^−ΔΔCT^ method.

### 2.4. Rapid Amplification of cDNA Ends (RACEs)

5′-RACE and 3′-RACE experiments were performed using a FirstChoice RLM-RACE kit (Thermo, Otsu, Japan) and a SMARTer RACE 5′/3′ kit (TakaRa, Otsu, Japan) according to the manufacturer’s protocols, respectively. Primer sequences of the RACE experiment are shown in Table S1. Total RNA isolated from porcine subcutaneous fat was used as templates in nested PCR reactions. The 5′- and 3′-RACE products were gel-purified using an Axygen AxyPrep DNA gel extraction kit (Axygen, Guangzhou, China) and cloned into the pEASY-blunt simple vector (Transgen, Beijing, China) following the manufacturer’s instructions, which were subsequently sequenced and analyzed.

### 2.5. Subcellular Localization

Cytoplasmic and nuclear RNAs from the preadipocyte cells were extracted using the PARIS kit (Invitrogen, AM1921, Thermo Fisher Scientific, Beijing, China). After obtaining total cellular RNA, nuclear, and cytoplasmic RNA components were separated and quantified, in which the same quantity of RNAs was used for reverse transcription and subsequent RT-qPCR analysis.

### 2.6. Cell Culture and Treatment

Porcine preadipocytes were cultured in the growth medium (89% porcine preadipocyte primary medium + 10% FBS + 1% penicillin–streptomycin solution). The cell differentiation medium was composed of 10% FBS, 89% porcine preadipocyte primary medium, 1% penicillin/streptomycin (Sigma-Aldrich, Cleveland, OH, USA), 1 μM dexamethasone (Sigma-Aldrich, USA), 10 μg/mL insulin (Gibco, Big Cabin, OK, USA), and 0.5 mM isobutylmethylxanthine (Sigma-Aldrich, Cleveland, OH, USA). To maintain the differentiation state of preadipocytes, 500 nM insulin was added to the cell growth medium. When the cells grew to about 80%, the differentiation medium was changed and induced for 4 days. The culture medium was changed every 48 h. When the cells began to appear as lipid droplets, they were replaced with a maintenance differentiation medium and continued to culture.

### 2.7. Cell Counting kit-8 Assay

Porcine preadipocytes were seeded in 96 well plates with a density of 3 × 10^3^ cells/well. After knockdown with *LincRNA-ROFM* for 48 h, CCK-8 kit (Solarbio, Beijing, China) was used to measure cell viability according to the kit instruction. Cell activity was quantified by measuring the absorbance of each hole at 450 nm.

### 2.8. RNA Oligonucleotides

siRNA molecules specifically targeting the mRNA of *LincRNA-ROFM* were purchased from *RIBOBIO* (Guangzhou, China) (*si-LincRNA-ROFM-1*: GCTTGAGACAGAAACATAA; si-*LincRNA-ROFM-2*: CGCACTGAATCACATTTGA; *si-LincRNA-ROFM-3*: CACAAGCAACGGAACAAGA). The negative control duplex, which was also provided by *RIBOBIO*, was not homologous to any mammalian genes and is widely used in knockdown assays. The *miR-133b* mimics, negative control (NC) mimics, *miR-133b* inhibitors, and NC inhibitors were all purchased from *RIBOBIO*.

### 2.9. Transfections

Porcine preadipocytes were transfected with a negative control duplex or siRNAs against *LincRNA-ROFM* using Lipofectamine 3000 Transfection Reagent (Thermo Fisher Scientific, Beijing, China) following the manufacturer’s instructions. Nucleic acids were diluted in Opti-MEM Medium (Gibco, Gaithersburg, MD, USA). All experiments were carried out at least three times independently.

### 2.10. Dual-Luciferase Reporter Assay

Wild-type or mutant *LincRNA-ROFM* and *AdipoQ*-3’UTR dual-luciferase reporter (200 ng) and *miR-133b* mimic or NC mimic (50 nmol/L) were co-transfected into preadipocytes. After 48 h transfection, cells were washed by PBS twice, and the activities of firefly and *Renilla* luciferase were measured according to the manual of Luc-pair Duo-Luciferase Assay Kit 2.0 (GeneCopoeia, Rockville, MD, USA). All the data were acquired by averaging the results from three independent repeats.

### 2.11. Adipocyte Lipid Accumulation

To assess the lipid accumulation in adipocytes, Oil Red O and triglyceride (TG) assays were performed, respectively. The cells were washed with PBS and fixed in 4% formaldehyde for 10 min. The cells were then stained with Oil Red O working solution (Solarbio, Beijing, China) according to the manufacturer’s manual. Images were acquired by inverted fluorescent microscope (Nikon, Tokyo, Japan) with a 20× objective lens (200× magnification) and quantified by Image J software (National Institutes of Health, Bethesda, MD, USA). For the triglyceride test, intracellular triglycerides were assayed using the triglyceride assay kit (GPO-POD; Applygen Technologies Inc., Beijing, China).

### 2.12. Detection of AdipoQ Concentration by Enzyme-Linked Immunosorbent Assay (ELISA)

The culture medium was centrifuged at 12,000 g at 4 °C for 10 min to separate the supernatants. The concentration of AdipoQ in the culture supernate was determined using an ELISA kit per the manufacturer’s instructions (Wuhan Huamei Biotechnology Co., Ltd., Wuhan, China).

### 2.13. Western Blot Analysis

Briefly, protein quantification was performed using a BCA Kit (Beyotime, Shanghai, China). Protein lysates were subjected to SDS-PAGE and subsequently electrotransferred to a polyvinylidene fluoride membrane (PVDF). Diluted with primary and secondary antibodies dilution of 1:2000–500, the membrane was incubated with the indicated primary antibodies (AdipoQ (ABclonal, Wuhan, China), GAPDH (ABclonal, Wuhan), AdipoR1(ABclonal, Wuhan), AdipoR2 (ABclonal, Wuhan)) overnight at 4 °C and HRP-conjugated secondary antibodies, incubated at 37 °C for 2 h, and then rinsed with TBST 3 times, followed by blot signals were visualized by eECL Western Blot Kit (CWbio, Taizhou, China) and views were recorded by Bio-Rad Gel Doc XR Gel Documentation System (Bio-Rad, San Francisco, CA, USA). The quantification was performed with Image J software.

### 2.14. Statistical Analysis

One-way ANOVA data from IBM SPSS statistics V.20.0 software (SPSS Inc., Chicago, IL, USA) was used for statistical analysis. The results were expressed as mean ± SEM. *p <* 0.05 means significant difference, and *p <* 0.01 or *p <* 0.001 indicates extremely significant difference. All quantitative experiments were repeated three times.

## 3. Results

### 3.1. Identification of LincRNA-ROFM as a Candidate lncRNA

**Figure 1:**
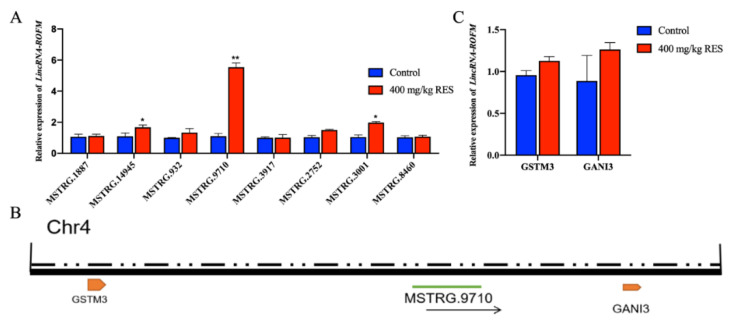
Identification of *LincRNA-ROFM* as a candidate lncRNA. (**A**) mRNA expression levels of candidate lncRNAs in porcine subcutaneous fat in control and 400 mg/kg RES groups as detected by RT-qPCR assay. (**B**) The map of lncRNA-MSTRG.9710 genome location model. Chr4, chromosome 4. (**C**) mRNA expression levels of *GSTM3* and *GNAI3* in control and 400 mg/kg RES groups as detected by RT-qPCR assay. Data are presented as the mean ± SEM (n = 3) vs. control, * *p* < 0.05, ** *p* < 0.01.

According to the results of RNA-seq of porcine subcutaneous fat of the control group and 400 mg/kg RES group, eight candidate lncRNAs were verified by RT-qPCR (Figure 1A). We found that the difference in *LncRNA-MSTRG.9710* was extremely significant between the two groups (*p <* 0.01), and the difference in the TPM value of *LncRNA-MSTRG.9710* was the largest in the sequencing results. Hence, *LncRNA-MSTRG.9710* was selected for subsequent verification. *LncRNA-MSTRG.9710* is located on chromosome 4 of porcine (Figure 1B), between the glutathione S-transferase m3 (*GSTM3*) and guanylate binding protein 3 (*GNAI3*) coding genes and belongs to intergenic lncRNA. Since lncRNAs can regulate the expression of adjacent genes, the expression levels of *GSTM3* and *GNAI3* genes in subcutaneous fat of pigs in the control group and RES group were detected by RT-qPCR. We found that there was no significant difference between the two groups (Figure 1C). To facilitate subsequent research and reporting, *LncRNA-MSTRG.9710* was named *LincRNA-ROFM* (long intergenic non-coding RNA, regulator of fat metabolism) according to its location and function.

### 3.2. LincRNA-ROFM Is Highly Expressed in Pig Liver and Affected by Fat Deposition and RES

**Figure 2:**
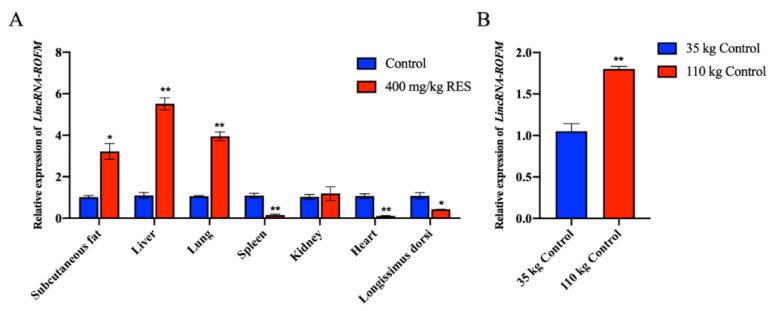
*LincRNA-ROFM* is highly expressed in pig liver and affected by fat deposition and RES. (**A**) The expression profiles of *LincRNA-ROFM* in porcine subcutaneous fat in control and 400 mg/kg RES groups as detected by RT-qPCR assay. The tissues corresponding to the control group were used as controls. (**B**) The expression profiles of *LincRNA-ROFM* in porcine subcutaneous fat in 35 kg and 110 kg control groups as detected by RT-qPCR assay. Data are presented as the mean ± SEM (n = 3) vs. control, * *p* < 0.05, ** *p* < 0.01.

The expression pattern of *LincRNA-ROFM* in different tissues was detected by RT-qPCR. The expression of *LincRNA-ROFM* was highest in pig liver in control and 400 mg/kg RES groups (Figure 2A). Since the liver is the center of fat metabolism, we speculated that *LincRNA-ROFM* might regulate fat metabolism. RT-qPCR of *LincRNA-ROFM* in subcutaneous fat showed that its expression was significantly higher in pigs weighing 110 kg than those weighing 35 kg (*p <* 0.01) (Figure 2B). These results indicated that the expression of *LincRNA-ROFM* varies with tissues, and is also affected by fat deposition and RES.

### 3.3. The Full-Length Sequence of LincRNA-ROFM Was Obtained by RACEs Technology

**Figure 3:**
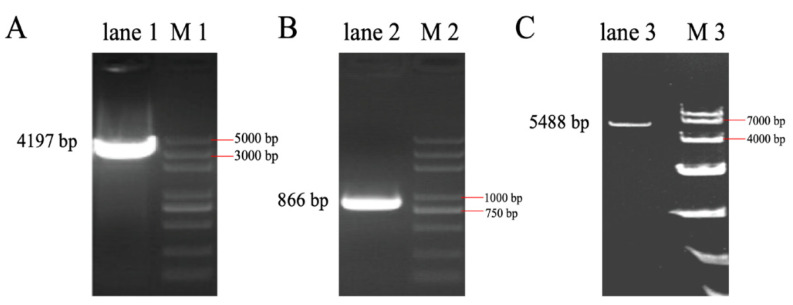
The full-length sequence of *LincRNA-ROFM* was obtained by RACE technology. (**A**) The result of 5′-RACE in lane 1. (**B**) The result of 3′-RACE in lane 2. (**C**) Agarose gel test to verify the full length of *LincRNA-ROFM* in lane 3. Note: M1: marker is DL 5000; M2: marker is DL 5000; M3: marker is DL 10,000.

5’RACE and 3’RACE PCR followed by nucleic acid gel electrophoresis are shown in Figure 3A, B, respectively. After sequencing, we found that the 5’ end expansion was 4197 bp (Figure 3A) and the 3’ end expansion was 866 bp (Figure 3B). After comparison with the initial sequence, it was confirmed that the full length of *LincRNA-ROFM* was 5488 bp. Gel electrophoresis showed that the fragment size was about 5400 bp (Figure 3C), indicating that the full-length lncRNA sequence was successfully obtained (Supplementary File S1).

### 3.4. LincRNA-ROFM Is Mainly Located in Cytoplasm of Porcine Preadipocytes and No Coding Ability

**Figure 4:**
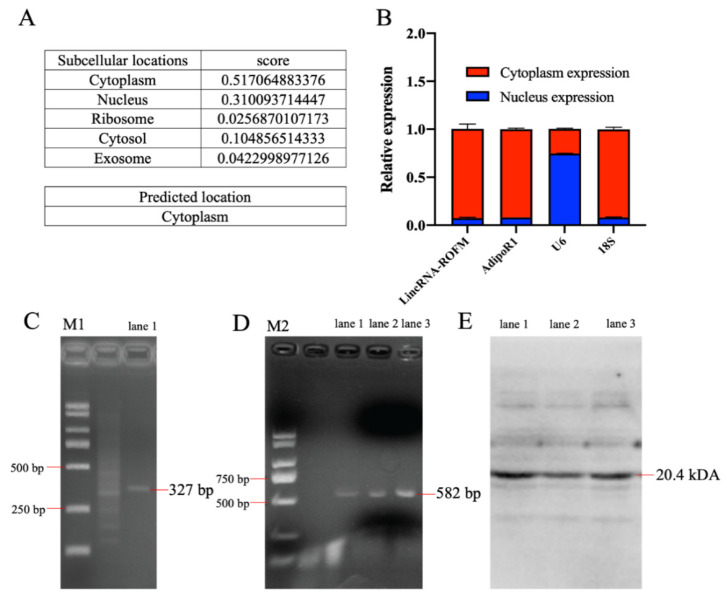
*LincRNA-ROFM* is mainly located in cytoplasm of porcine preadipocytes and no coding ability. (**A**) The subcellular localization of *LincRNA-ROFM* by *Lnclocator* online software. (**B**) The relative expression level of *LincRNA-ROFM* in cytoplasm and nucleus of porcine preadipocytes by RT-qPCR. *U6* as an internal reference in the nucleus, *18S* was used as a cytoplasmic reference and *AdipoR1* was used as a negative control. (**C**,**D**) The results of *LincRNA-ROFM* ORF detected by Agarose gel detection PCR. Note: *LincRNA-ROFM* ORF1 is 327 bp (**C**); *LincRNA-ROFM* ORF2 is 582 bp (**D**), and marker is DL 2000. (**E**) The coding ability of *LincRNA-ROFM* detected by WB. Note: lane 1: *pET-32a*; lane 2: *LincRNA-ROFM ORF1* and lane 3: *LincRNA-ROFM ORF2*.

Lnclocator (http://www.csbio.sjtu.edu.cn/bioinf/lncLocator/, accessed on 13 April 2021), an online software, predicted that *LincRNA-ROFM* was mainly located in the cytoplasm (Figure 4A). RT-qPCR of nuclear and cytoplasmic fractions indicated that *LincRNA-ROFM* is predominantly localized in cytoplasm of preadipocytes (Figure 4B). Therefore, according to the software prediction and experimental analysis results, *LincRNA-ROFM* was mainly located in cytoplasm of porcine preadipocytes.

To assess whether *LincRNA-ROFM* has a protein-coding ability, the coding potential calculator (CPC) (http://cpc2.gao-lab.org/, accessed on 1 August 2021), an online software, was used in the present study. The prediction showed that *LincRNA-ROFM* does not have coding potential. Next, to verify whether *LincRNA-ROFM* has the ability to encode small peptides. We found that *LincRNA-ROFM* has two ORFs (position: ORF1: 1917–2240 bp, ORF2: 3801–4382 bp) with lengths more than 200 bp through the NCBI-ORF online website (https://www.ncbi.nlm.nih.gov/orffinder/, accessed on 25 August 2021). The two sequences were amplified by PCR and cloned into prokaryotic expression vector pET-32a, and the sequencing results were correct (Figure 4C,D). The expression of foreign fragments in the bacterial solution was induced by IPTG, and proteins in the bacterial solution were extracted. The fusion tag of prokaryotic expression vector pET-32a was about 20.4 kDa. It was predicted that the size of the ORF1 small peptide was 12.11 kDa and that of the ORF2 small peptide was 20.19 kDa. WB results are shown in Figure 4E. The size of the empty bacterial solution and the protein fragments of the two groups of imported foreign fragments were the same, both around 20 kDa, and there were no clear bands in other positions. Therefore, those showed that *LincRNA-ROFM* presents no coding ability.

### 3.5. LincRNA-ROFM Knockdown Promoted the Proliferation and Differentiation of Porcine Preadipocytes

**Figure 5:**
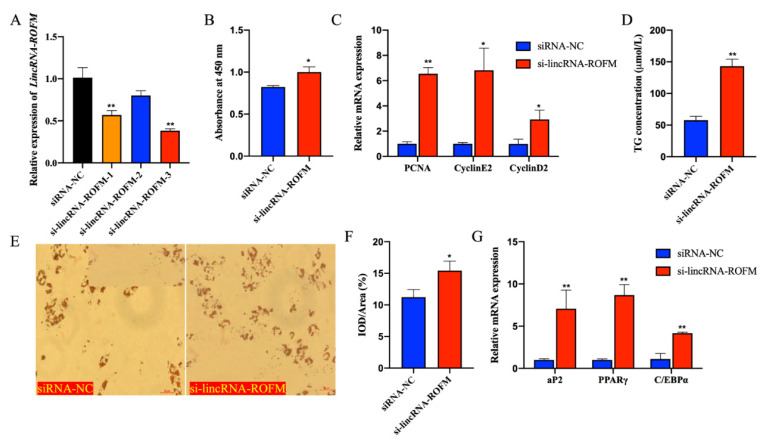
*LincRNA-ROFM* knockdown promoted the proliferation and differentiation of porcine preadipocytes. (**A**) The relative expression levels of *LincRNA-ROFM in* porcine preadipocytes transfecting with siRNA 1, 2, and 3. (**B**) Proliferation of porcine preadipocytes following transfection with *LincRNA-ROFM* siRNA after 48 h detected by CCK8 assay. (**C**) mRNA expression levels of *PCNA*, *cyclinE2,* and *cyclinD2* in porcine preadipocytes transfected with *LincRNA-ROFM* siRNA after 48 h detected by RT-qPCR assay. (**D**) After extracting Oil Red O with isopropanol, the OD value (510 nm) was detected following transfection with *LincRNA-ROFM* siRNA after 6 days. (**E**,**F**) Photomicrographs and quantitative of Oil Red O showed transfection with *LincRNA-ROFM* siRNA increased the lipid accumulation after 6 days. Scale bar, 50 mm. (**G**) mRNA expression levels of *PPARγ*, *aP2,* and *C/EBPα* in porcine preadipocytes transfected with *LincRNA-ROFM* siRNA after 6 days as detected by RT-qPCR assay. Data are presented as the mean ± SEM (n = 3) vs. siRNA-NC, * *p* < 0.05, ** *p* < 0.01.

To explore the functions of *LincRNA-ROFM*, we performed loss-of-function experiments of *LincRNA-ROFM* in porcine preadipocytes. *si-LincRNA-ROFM-1, si-LincRNA-ROFM-2, si-LincRNA-ROFM-3,* and si-NC were transferred into preadipocytes, respectively. After 48 h, the cells were collected. The knockdown efficiency was detected by RT-qPCR. The results showed that siRNAs specific to *si-LincRNA-ROFM-3* reduced their RNA level by about 60% (Figure 5A). Therefore, *si-LincRNA-ROFM-3* was used to knock down the expression of *LincRNA-ROFM* in subsequent experiments.

To explore the function of *LincRNA-ROFM* in adipocyte proliferation, after the knockdown of *LincRNA-ROFM* for 48 h, the cells were collected, and the changes in expression of cell proliferation genes were detected by RT-qPCR. We found that the viability of porcine preadipocytes increased significantly after the knockdown of *LincRNA-ROFM* (*p <* 0.05) by CCK8 assay (Figure 5B). In addition, the expression of *PCNA*, *Cycline2* and *Cyclind2* genes were downregulation significantly after knockdown of *LincRNA-ROFM* (*p <* 0.05 or *p <* 0.01) (Figure 5C). These results suggested that *LincRNA-ROFM* can inhibit adipocyte proliferation.

Next, we explored the function of *LincRNA-ROFM* in the differentiation of adipocytes. Six days after, porcine preadipocytes were induced to differentiate. Oil Red O staining suggested that knockdown of *LincRNA-ROFM* promoted adipogenesis (*p* < 0.05) (Figure 5E,F). The determination of triglycerides (TGs) in preadipocytes showed that the accumulation of TGs in preadipocytes increased significantly after the knockdown of *LincRNA-ROFM* (*p* < 0.01) (Figure 5D). RT-qPCR results founded that the expression of *PPARγ*, *aP2*, and *C/EBPα* increased significantly after *LincRNA-ROFM* knockdown (*p* < 0.01) (Figure 5G). The above results showed that *LincRNA-ROFM* knockdown promotes adipocyte differentiation, which in turn indicates that *LincRNA-ROFM* inhibits adipocyte differentiation.

### 3.6. LincRNA-ROFM Regulates the Expression of AdipoQ

**Figure 6:**
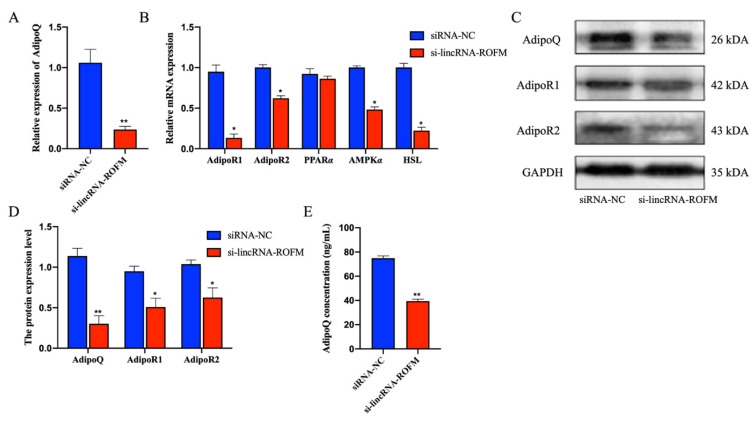
*LincRNA-ROFM* regulates the expression of *AdipoQ*. (**A**) mRNA expression level of *AdipoQ* in porcine preadipocytes transfected with *LincRNA-ROFM* siRNA after 6 days as detected by RT-qPCR assay. (**B**) mRNA expression levels of *AdipoR1*, *AdipoR2, PPARα, AMPKα,* and *HSL* in porcine preadipocytes transfected with *LincRNA-ROFM* siRNA after 6 days as detected by RT-qPCR assay. (**C**) Western blot of porcine preadipocytes transfected with *LincRNA-ROFM* siRNA after 6 days using antibodies against AdipoQ, AdipoR1, and AdipoR2. (**D**) Histogram bar graph after Western blot signal quantification indicating the protein level of AdipoQ, AdipoR1, and AdipoR2 after transfection with porcine preadipocytes transfected with *LincRNA-ROFM* siRNA after 6 days. (**E**) The concentration of AdipoQ in porcine preadipocytes transfected with *LincRNA-ROFM* siRNA after 6 days as detected by ELISA assay. Data are presented as the mean ± SEM (n = 3) vs. siRNA-NC, * *p* < 0.05, ** *p* < 0.01.

Next, we turn our attention to *AdipoQ*, a critical regulator of adipocyte differentiation. To explore whether the *AdipoQ* gene was regulated by *LincRNA-ROFM,* we detected *AdipoQ* mRNA and protein expressions in adipocytes with *LincRNA-ROFM* knockdown in adipocytes. The results indicated that AdipoQ and AdipoQ signaling pathway-related genes *AdipoR1*, *AdipoR2*, *AMPKα,* and *HSL* decreased significantly (*p* < 0.05 or *p* < 0.01) (Figure 6A–D). Similarly, ELISA results showed that the concentration of AdipoQ decreased in cells after the knockdown of *LincRNA-ROFM* (Figure 6E). The expression of the AdipoQ gene is positively correlated with the expression level of *LincRNA-ROFM*. Based on the above results, it can be proved that *LincRNA-ROFM* positively regulates the expression of AdipoQ.

### 3.7. LincRNA-ROFM Acts as a Molecular Sponge for miR-133b and Regulates the Expression of AdipoQ

**Figure 7:**
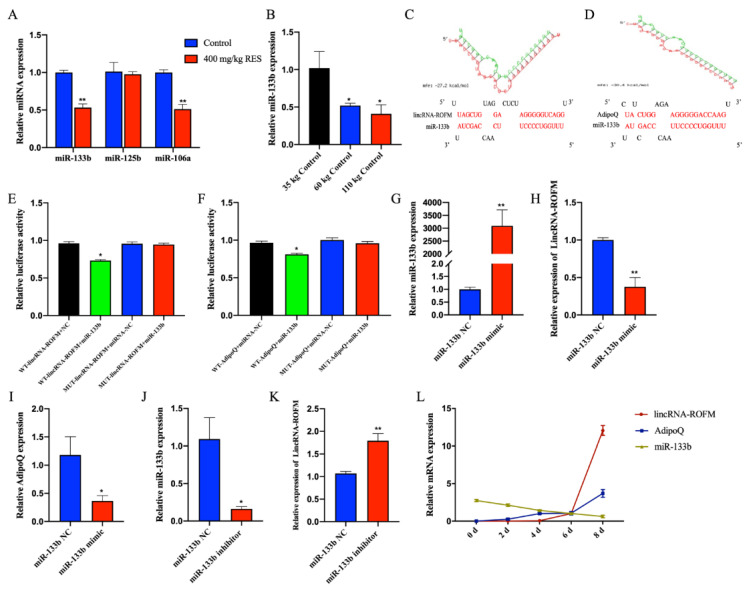
*LincRNA-ROFM* acts as a molecular sponge for *miR-133b* to regulate the expression of *AdipoQ*. (**A**) The expression profiles of *miR-133b*, *miR-125b,* and *miR-106a* in porcine subcutaneous fat in control and 400 mg/kg RES groups as detected by RT-qPCR assay. (**B**) The expression profiles of *miR-133b* in porcine subcutaneous fat in 35 kg, 60 kg, and 110 kg control groups as detected by RT-qPCR assay. (**C**) Complementary pairing between the sequences of *LincRNA-ROFM* and *miR-133b*. The above scheme shows the calculated ΔG value. (**D**) Complementary pairing between the sequences of *miR-133b and AdipoQ*. The above scheme shows the calculated ΔG value. (**E**) *LincRNA-ROFM* wild or mutant type psi-check2 plasmid was transfected into porcine preadipocytes together with miR-133b mimic. Luciferase activities were measured 24 h after transfection. (**F**) *AdipoQ* wild or mutant type psi-check2 plasmids were transfected into porcine preadipocytes together with *miR-133b* mimic. Luciferase activities were measured 24 h after transfection. (**G**) mRNA expression level of *miR-133b* in porcine preadipocytes after transfected with *miR-133b* mimics as detected by RT-qPCR assay. (**H**) mRNA expression level of *LincRNA-ROFM* in porcine preadipocytes after transfected with *miR-133b* mimics as detected by RT-qPCR assay. (**I**) mRNA expression level of *AdipoQ* in porcine preadipocytes after transfected with *miR-133b* mimics as detected by RT-qPCR assay. (**J**) mRNA expression level of *miR-133b* in porcine preadipocytes after transfected with *miR-133b* inhibitor as detected by RT-qPCR assay. (**K**) mRNA expression level of *LincRNA-ROFM* in porcine preadipocytes after transfected with *miR-133b* inhibitor as detected by RT-qPCR assay. (**L**) mRNA expression levels of *LincRNA-ROFM, miR-133b,* and *AdipoQ* at different stages of preadipocyte differentiation (0, 2, 4, 6, and 8 d) as detected by RT-qPCR assay. Data are presented as the mean ± SEM (n = 3) vs. control, 35 kg control or NC, * *p* < 0.05, ** *p* < 0.01.

*LincRNA-ROFM* and *AdipoQ* are located on different chromosomes, while *LincRNA-ROFM* is mainly located in cytoplasm. We speculated that *LincRNA-ROFM* can regulate *AdipoQ* through the known mechanism of ceRNA, that is, *LincRNA-ROFM* competitively “sponges” miRNA, resulting in the activation of its target gene *AdipoQ*. Among the miRNAs predicted by the RNAhybrid (https://bibiserv.cebitec.uni-bielefeld.de/rnahybrid/, PITA (http://www.pita.org.fj) and MIRDB (http://mirdb.orghttp://mirdb.org, accessed on 14 November 2021), miRNAs related of fat metabolism, including *miR-133b*, *miR-125b,* and *miR-106a* were screened. The RT-qPCR result showed that *miR-133b* and *miR-106a* were downregulated significantly in the porcine subcutaneous fat of the RES group (*p* < 0.01) (Figure 7A). Rnahybrid predicted the binding sites between the miRNA and *LincRNA-ROFM*. We found that there were highly reliable binding sites between *miR-133b* and *LincRNA-ROFM* (Figure 7C). Interestingly, we noticed that *miR-133b* and *AdipoQ* have a putative binding site (Figure 7D). In addition, the RT-qPCR result of *miR-133b* in subcutaneous fat of 35 kg, 60 kg, and 110 kg pigs showed that the expression level decreased with the increase in pig weight and fat deposition (*p* < 0.05, Figure 7B), and it was just a result cross to *lincRNA-ROFM*. Therefore, we speculated that *miR-133b* is involved in the regulation of the adiponectin pathway.

Next, we confirmed that *LincRNA-ROFM* indeed targets *miR-133b*. We constructed wild-type, mutant *LincRNA-ROFM* and mutant *AdipoQ*, and luciferase reporters containing a wild-type or mutant target site from *LincRNA-ROFM* and *AdipoQ*. Our results showed that the relative luciferase activity of *LincRNA-ROFM*-wild and *AdipoQ* were reduced significantly upon co-transfection with *miR-133b* mimic, compared with the negative controls, while the downregulation of *miR-133b* significantly enhanced the relative luciferase activity (Figure 7E,F). This suggested that *LincRNA-ROFM* binds *miR-133b* and that *AdipoQ* is targeted by *miR-133b*. Based on these results, we verified that *LincRNA-ROFM* may function as a ceRNA, leading to the liberation of the corresponding miRNA-targeted (*AdipoQ*) transcripts.

We further verified the possible effects of *LincRNA-ROFM* as a molecular sponge for *miR-133b* to regulate the expression of *AdipoQ*. We then transfected *miR-133b* mimics or inhibitors into preadipocytes. As expected, *miR-133b* overexpression strongly decreased the mRNA levels of *LincRNA-ROFM* and *AdipoQ* (Figure 7G–I), while *miR-133b* inhibitors increased the mRNA levels of *LincRNA-ROFM* (Figure 7J,K). These results agreed with the ceRNA hypothesis that *LincRNA-ROFM* inhibits *miR-133b* function, leading to increased expression of their target gene *AdipoQ.*

Furthermore, we measured the expression of *LincRNA-ROFM*, *miR-133b,* and *AdipoQ* at different stages of preadipocytes differentiation (0, 2, 4, 6, and 8 d). Those results showed that *LincRNA-ROFM* and *AdipoQ* were not expressed before cell differentiation, and their expression levels increased sharply in the differentiation medium for 8 days. In contrast, the expression of *miR-133b* decreased gradually during preadipocyte differentiation (Figure 7L). This again showed that *LincRNA-ROFM* has a synergistic effect with *AdipoQ* expression, which was opposite to *miR-133b.*

### 3.8. RES Can Regulate the Expression of LincRNA-ROFM by Regulating PPARa and C/EBPa

**Figure 8:**
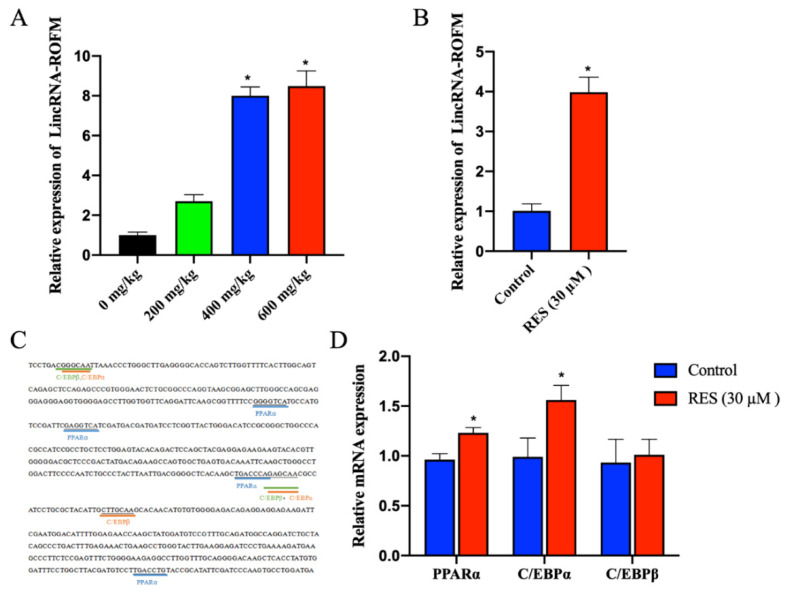
RES can regulate the expression of *LincRNA-ROFM* by *PPARα* and *C/EBPα*. (**A**) The expression profiles of *LincRNA-ROFM* in porcine subcutaneous fat in 0 mg/kg, 200 mg/kg, 400 mg/kg, and 600 mg/kg RES groups as detected by RT-qPCR assay. (**B**) The expression profiles of *LincRNA-ROFM* in porcine preadipocytes treated with RES (30 µM) as detected by RT-qPCR assay. (**C**) Predicting the transcription factor binding site on the *LincRNA-ROFM* promoter region. (**D**) mRNA expression levels of *PPARα*, *C/EBPα,* and *C/EBPβ* in porcine preadipocytes treated with RES (30 µM) as detected by RT-qPCR assay. Data are presented as the mean ± SEM (n = 3) vs. control, * *p* < 0.05.

To further understand the potential regulatory mechanism of RES on *LincRNA-ROFM*. The RT-qPCR results of *LincRNA-ROFM* in subcutaneous fat of pigs fed with 0 mg/kg, 200 mg/kg, 400 mg/kg, and 600 mg/kg RES showed that *LincRNA-ROFM* increased with an increase in RES in a dose-dependent manner (Figure 8A). The expression level of *LincRNA-ROFM* in the 400 mg/kg of RES group was higher significantly than that of the 0 mg/kg and 200 mg/kg groups (*p* < 0.05). After preadipocytes were treated with RES (30 µM), we found that the expression level of *LincRNA-ROFM* increased significantly (*p* < 0.05) (Figure 8B). These results showed that RES can promote the expression of *LincRNA-ROFM.*

Moreover, to explore the RES regulation mechanism of *LincRNA-ROFM*, Bioinformatic analysis found that there were multiple transcription factor binding sites 200–400 bp upstream of *LincRNA-ROFM*, including *PPARα*, *C/EBPα*, *C/EBPβ* involved in fat metabolism (Figure 8C). RT-qPCR results showed that the expression level of *PPARα* and *C/EBPα* were upregulated significantly after adding RES in preadipocytes (Figure 8D). Therefore, it is speculated that RES can regulate the expression of *LincRNA-ROFM* by *PPARα* and *C/EBPα*.

## 4. Discussion

**Figure 9:**
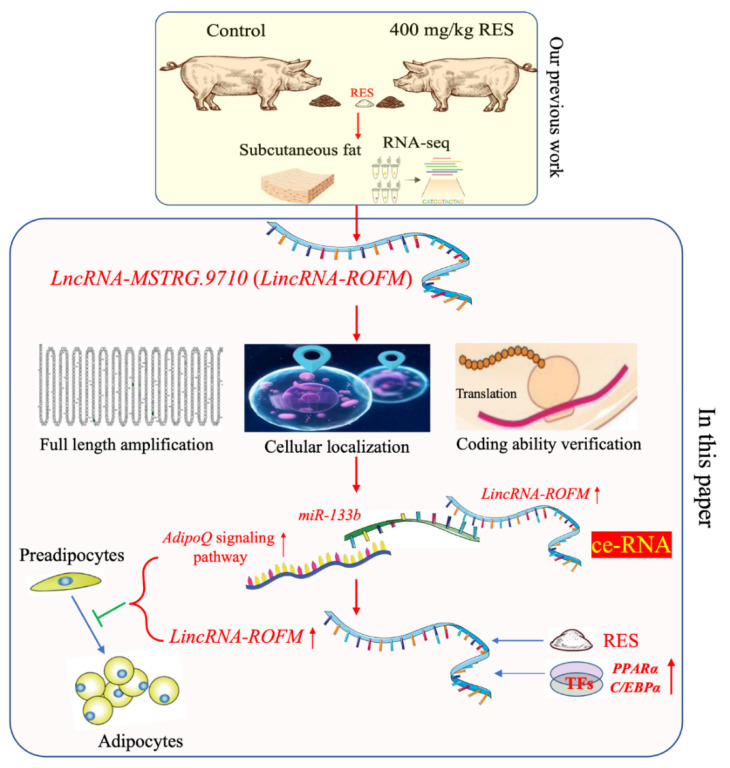
Study routes and block diagram. The *LincRNA-ROFM* were found in pig subcutaneous fat between control group and 400 mg/kg RES group by RNA-seq on the basis of previous work in our group. In this paper, we verified the functional mechanism of RES to reduce the thickness of porcine subcutaneous fat by *LincRNA-ROFM* through molecular and cell biological experiments.

In this study, we combine nutrient factors with detailed mechanistic studies to describe an adipocyte proliferation and differentiation related lncRNA, *LincRNA-ROFM*, which functions as a ceRNA to inhibit subcutaneous fat deposition by regulating the expression of the target gene of *miR-133b* and is regulated by RES and transcription factors *PPARα* and *C/EBPα* (Figure 9). The RES/LincRNA-ROFM/miR-133b/AdipoQ regulatory network preliminarily explains the mechanism of action of RES in inhibiting fat deposition, providing new insight into the downstream mechanisms of RES by regulating the lncRNA. To the best of our knowledge, this was the first study concerning the RES regulation of fat deposition through lncRNA regulated *AdipoQ* pathway in pigs. Our study provides new insight into the underlying mechanism of fat deposition regulation by pig molecular nutriology and may also contribute to improving pork quality.

Previous studies showed RES can improve glucose metabolism and lipid profile, decrease fat deposition and steatosis [37], and inhibit the occurrence of obesity [38]. In addition, it has been reported that daily high doses of RES in rats are not harmful to body weight, and food and water consumption [39,40]. Therefore, RES can be considered as a feed additive for producing high-quality (high lean meat percentage) pork. In our previous study, we observed that the dietary RES supplementation at doses of 200, 400, and 600 mg/kg/d had no significant effects on growth, food intake, or average food intake in finishing pigs, respectively, which is consistent with the previous study of mice [25,41]. Interestingly, we found that backfat thickness was lower significantly in the RES group as compared to the control group. This is in agreement with the theory that RES can inhibit the occurrence of obesity [42,43]. Compared with mice, pigs are very similar to humans in anatomy, physiology, and genetics and it has become the most valuable and ideal disease model animal in the research of life science and medical science [44,45]. Therefore, elaborating the specific molecular mechanism of RES reducing pig backfat thickness can not only promote RES as a food-derived feed additive to produce high-quality pork but also lay a theoretical foundation for promoting pigs as an animal model of obesity.

According to a recent study, phytochemicals can regulate biological processes through epigenetics, including lncRNA, miRNA, and DNA methylation [46]. For instance, RES can improve high-fat diet-induced insulin resistance in mice by downregulating the *lncRNA NONMMUT008655.2* [36]. In our previous study, we performed the lncRNA sequencing of porcine subcutaneous fat in the control group and 400 mg/kg RES group and found some lncRNAs related to adipogenesis, including *LincRNA-ROFM*, a novel lncRNA. However, it is unclear whether RES can inhibit fat deposit by regulating *LincRNA-ROFM*. In the present study, we identified that the expression level of *LincRNA-ROFM* was the highest in pig liver and it was affected by fat deposition and RES, which is consistent with characteristics of lncRNAs in the tissue-specific expression [47]. These results indicate that RES may regulate fat deposition by regulating the expression of *LincRNA-ROFM.*

To identify the function of *LincRNA-ROFM*, we used small interference performed *LincRNA-ROFM* loss-of-function experiments in porcine preadipocytes. The CCK-8 assay and cell proliferation marker genes (*PCNA*, *cyclinE2*, *cyclinD2*) detection indicated that *LincRNA-ROFM* can inhibit adipocyte proliferation. Oil red-O staining, contents of TG, and the cell differentiation marker gene detection indicated that *LincRNA-ROFM* can inhibit adipocyte differentiation. Adipocyte proliferation and differentiation are the keys to adipogenesis [48]. These results further suggest that *LincRNA-ROFM* plays a vital role in porcine fat deposition. While lncRNAs do not encode proteins, certain aspects of lncRNA biology parallel that of mRNA (coding small peptide) [49]. In addition, lncRNA functions in different subcellular compartments are directed by local molecular interactions [50]. We first have determined the *lincRNA-ROFM* located in the cytoplasm of porcine preadipocytes and no coding ability.

In search of the possible downstream effector of *LincRNA-ROFM* mediated regulation of porcine preadipocyte proliferation and differentiation, we focused on *AdipoQ*, a critical regulator of the adipocyte differentiation [51]. *AdipoQ* can increase the activity and expression of enzymes that promote triglyceride hydrolysis and promote fatty acids decomposition to reduce the materials for synthesizing triglycerides [52]. In addition, *AdipoQ* can bind to AdipoR1 and *AdipoR2* to activate *AMPK* and *PPARα* signaling factors to promote fatty acid oxidation and inhibit lipid synthesis and thereby exerting lipid-lowering effects [53]. These reports emphasized that AdipoQ secretion negatively correlates with adipose tissue deposition [35,54]. In our study, we found that *LincRNA-ROFM* positively regulates the expression of AdipoQ. Subsequent bioinformatic analyses and dual-luciferase reporter gene assays indicated that *LincRNA-ROFM* is a direct target of *miR-133b* and *miR-133b* is a direct target of *AdipoQ*. Previous studies demonstrated that miR-133b can improve glucose tolerance, and insulin sensitivity, and decreases plasma levels of triglycerides [50]. Therefore, we speculate that *LincRNA-ROFM* may act as a ceRNA in the regulation of porcine fat deposition. Interestingly, *miR-133b* negatively regulated *LincRNA-ROFM* and AdipoQ expression in porcine preadipocytes with different differentiation days. These results further demonstrate that *LincRNA-ROFM* “sponges” *miR-133b* to inhibit porcine fat deposition. *LincRNA-ROFM* communicates with and regulates *AdipoQ* by direct competition for miR-133b binding, this is a typical mode of competing for endogenous RNAs (ceRNAs) [55]. In addition, we found that RES can promote the expression of *LincRNA-ROFM* by *PPARα* and *C/EBPα* in vitro experiments. This may indicate phytochemicals can regulate biological processes through lncRNA [56]. *PPARα* can promote fatty acid β-oxidation and plays an important role in the regulation of lipid metabolism [57,58]. *C/EBPα* is an adipogenic markers gene associated with lipid composition and distribution [59,60]. The RES/*LincRNA-ROFM/miR-133b/AdipoQ* regulatory network preliminarily explains the mechanisms of action of RES inhibiting fat deposition.

## 5. Conclusions

**Figure 10:**
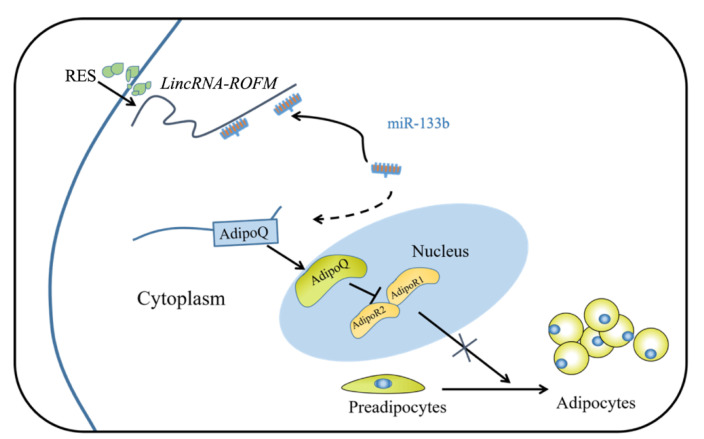
Schematic representation of the RES inhibition of subcutaneous fat deposition in pigs. RES can upregulate the expression of *LincRNA-ROFM*. *LincRNA-ROFM* communicates with and regulates AdipoQ by directly competing for miR-133b binding to inhibit adipocyte proliferation and differentiation.

RES increases *LincRNA-ROFM* that sponges miR-133b and thereby upregulates the expression of its target gene, that is, AdipoQ. Hence, RES serves as an essential feed additive to improve pork quality (Figure 10).

## Data Availability

Data are available on request from the author(s). The datasets generated during the experiment that support the findings of this study are available from the corresponding authors upon reasonable request.

## Supplementary Materials

The following supporting information can be downloaded at: https://www.mdpi.com/article/10.3390/foods11172690/s1, Table S1: Primers for RACE experiments. Table S2: Specific primers used for RT-qPCR. File S1: Full-length sequence of *LincRNA-ROFM*.
