# Supplementary material for: Resveratrol Inhibits Proliferation and Differentiation of Porcine Preadipocytes by a Novel LincRNA-ROFM/miR-133b/AdipoQ Pathway
Source: Foods. 2022 Sep 3;11(17):2690. doi: 10.3390/foods11172690 (PMC9455634; doi:10.3390/foods11172690)
Supplement: Supplementary file 1 [file foods-11-02690-s001.zip › foods-1816393-supplementary.pdf]

foods

Supplementary Materials

Resveratrol inhibits proliferation and differentiation of porcine preadipocytes by a novel *LincRNA-ROFM/miR-133b/AdipoQ* pathway

Qinyang Jiang [1,†], Sanbao Zhang [1,†], Xiaotong Gao [1], Yan Hu[1], Yu Zhang[1], Yujian Shen[1], Yuhang Jiang[1] and Yanna Huang [1,*]

1 College of Animal Science and Technology, Guangxi University, Nanning Guangxi 530004, China
* Correspondence: huangyn@gxu.edu.cn (Y.H.)
† The authors contributed equally to this work.

Table S1. Primers for RACE experiments.

Primer name	Primer sequence
rMSTRG.9710-F1	5'-CCCTCAAAGCGGCTGAGGAAGGCTTT-3'
rMSTRG.9710-F2	5'-GGGATCAAATATGCGGTACAGGTCAAG-3'
rMSTRG.9710-R1	5'-CTTCCTTTCTGTGCTTGCTCCTGCTGTGA-3'
rMSTRG.9710-R2	5'-GGGCTCAGTCCTGAGCTGCCCAG-3'

Table S2. Specific primers used for RT-qPCR.

Sequence definition	Sense primer (5'→3')	Antisense primer (5'→3')	Product length (bp)
MSTRG.1887	GCTGCCTCATTTGTTTCATCACC	GGCCCAACCAGAATACCCTA	187
MSTRG.3001	TTTGGAGCAGGTGCTTTCTG	CTGCTGCCAAGGTTCCAGTA	76
MSTRG.2752	CACTGGAGCAATAAGCCCCT	TCCGGTATGAACACTTGAGC	130
MSTRG.8460	CATGCACTTGGATGAAAACCG	TGAATCCGAAGCAGTAGCCTT	177
MSTRG.932	ATTAGAGCGGCTTTCCTTCCG	GGCTCTGAGAAGACAAGCTCA	163
MSTRG.14945	AAACTCCCTGCCCTTCGCTT	ACTGCTGCTTCTCAGGGATAG	134
MSTRG.3917	TGGAGACAGCTCTGCGAATC	TGGAGGACCGTCCAAAGTCA	155
MSTRG.9710	CACAGAAAGGAAGGCTTTCAGG	TTCCTGGCTTACGATGTCCTT	90
GSTM3	GGTTACTGGGATATTCGCGGG	GAGCTTGTTCTTCCCGTCCA	195
GNTI3	ATGGGGAGAAAGCGGCGAAA	AGCCGTCCCATGGCTCTAA	196
lincRNA-ROFM ORF1	gtggtggtggtggtgctcgagATGAGCTCTTCACAAC CCAGGA	tcgagctccgtcgacaagcttCTAAGCCTTTCCCAG GCTGC	327
lincRNA-ROFM ORF2	gtggtggtggtggtgctcgagATGCGCAGCCAAAATC CTAG	tcgagctccgtcgacaagcttTCAGCACCTCTGTAA TGTCTGGG	582
PCNA	GCATGGACTCGTCTCATGTCT	TTGGACATGCTGGTGAGGTT	115
cyclin D2	GTGCTGGGCAAGTTGAAGTG	GCGAACTTGAAGTCAGTGGC	158
cyclin E2	GAGCGGTAGCGGGTCTG	GCTTCTTGAGGGGAATCCGT	120
PPARγ	CCGAGAGCTGATCCAATGGT	GAACCCCGAGGCTTTATCCC	198
aP2	AGTTCCAGTTCCTCCCAAATC	CGTCTCTGAACTTCCCCCAC	168
C/EBPα	GGCATCTGCGAACACGAGAC	CCGGCTGTGCTGAAACAGGT	99
U6	TGGAACGCTTCACGAATTTGCG	GGAACGATACAGAGAAGATTAGC	87
AdipoQ	CGAGGTGGTCAAGGCTAAG	CAATGGCGTGGAGAAATAC	172
C/EBPα	GGCATCTGCGAACACGAGAC	CCGGCTGTGCTGAAACAGGT	99
C/EBPβ	CGACAGTTCGGAGCCAGGAA	TCCGCCTCGTAGTAGAAGTTG	99
HSL	CTTTGCGGGTATTCGGGAACA	ATGCTGCGGCGGTTGGA	192
PPARα	CATCCTCGCGGGAAAGG	GGCCATACACAGTGTCTCCATGT	70
AMPKα	AACATGGACGGGTTGAAGAG	CGCAGAAACTCACCATCTGA	193
AdipoR2	CCTCTTACAAGCCCACC	AGTCAGGCAGCACATCG	101
AdipoR1	CGAGGTGGTCAAGGCTAAG	CAATGGCGTGGAGAAATAC	172
18S	CCCACGGAATCGAGAAAGAG	TTGACGGAAGGGCACCA	122

File S1. Full length sequence of *LincRNA-ROFM*.

CGCTGGAAAGAGGAGCTTCCGCGCGCAGAGGCTGAGGGCTGCTCGCTTCCCGCCCACACTCGGACCTTGGCTCTGGCGGG

GAGTTGGCCCTCCGCGCTCGCTGGGCAAACAAGCCAGAAGCTGAACAAAGGCAGGACTCTTTCTCTTCCCTTCCCTTCCCT

TCCCTGAGGGCGTCATTTAGCCTCGTTCCCTTCAGGCTCTGACTGCCAACTTCAGGCCAAGGAATGGAGACCTGAAATCGG

CCCCTGTTTCAGGAGGAGCAGGACCCTCTCCCTGCATTTGGGAATCCGTGACAGCTGCGGTCCTCCTGAGCAGACCATCCG

TGGGAGGGGAAGAAACGCCCAGGTGTCCTATGATGATGTCCTGTCATCTGTGACCTGTGGTACACTCTGCACAAGAACAG

TGTCCTAGCTGCCAGCATTCCTCTTTGAAGGCTCAAGATTATGTTAGAACCAGGACAACAGGACATAGGTTGGTCTTTGGT

TCCTTTTCTCTGTGTAGAATATTTTACAAGGTGGTAGGTATATGAAGATAGATGAGGCGTTCCCATCATGGCTGGGTGGTTA

ACAAACCCGACTAGTATCCAGGAGGACCCAGGTTTGATCCCTGGCCTTTCCCAGTGGGTTAAAGATCCAGAGTTGCCTTGA

GCTGTGTGGTGTAGGTCGCAGGCGCAGCTCACATCTGGCGTTGCTGTGGCTGTGGTGTAGGCCTGTGGCTACAGCTCCGCG

ATTCGACCCCTAGCCTGGGAACCTCCATAGGCCACAGGGGCGGCCCTAAAACGACAAAAGACAAAAAAAAAAAAGAGA

AAAAAGAAAAAAACAGATGAGAGGAACTTTAAAAAACCAAATGGCTTTTACAAGTTAAAATGCAAGGTTGGTAAACTAC

AGAAAAATAAGCTTACCATGAGCTCACACTTGATACACTTGATAAGGAAAGAAAGGTATGAACCTAGTATTCAATGGAG

GCATGGGGGGTCGAGAAGGGAGACTTTAGCTAATCCCTGTGAAATCCCAGACTCAGAGTGTGCCCATCTCTGCTAAGTGTT

GGGGCTGGGAGGGTCTGTCACCCAAGACGTGCAGAGCTCAGTCCCTGTGGCAGGTGCTCCCCAGCCGTGCTGACCTTGCT

GGTCCTCGGGGCTCCAAGCTGAGGGTCCAGACTCAGGATGCAGCTCAAGGTCAGGTGGATTCCCTGATCAGTTTGCCCAC

AGGGAGGCAACGGTGGCCTCGCAAACATACACGGGCTTCACCCCATACCTCCTTATGGTTTCCATAGCTCATGAAGGAGT

TTTCATGCTGAGTGTCATACATCTTCAGCTTGAGACAGAAACATAACTGGGTTCCATGGAGAAAATGGCTTTTCTGACTAA

CATTAAAGAGAATACCAACATTGAAAATAGGTAGAACAGATACTGTAGTGTCAGGATTCCTGCATCCACTTGTTCCTTTTT

TGGAAAAAACAGTGACTGAAATCCTGCCATAGAGCAGGCACTGTGAGTAAGACCATCCTGCGTAATAAAGCCTGGAAGT

CAGCTGGGAAAGTCAAGGAGAGACAAACCACACTGGATCTACAGGAAAAGACTGGAACACATCCGGTAACTATCTAGA

GTCTGTGGGCAGGTGGATGGCCGATATGTACAGATTTTTAAGTGAAATGTTAAGGGAGAAAAGAGCAGAACCTGAGAGTG

CATGCACACACCCATTACATCTGCGCACAAGTGGGCATGAATGAAGATCAAACGAATATTTGAAGTGACCCCCCCACCCA

GAGCAAGTATAATTGGCTTAAGAGCTCTACCTTGAGCACCCACAGTCTCCCAGGGGCTGTTCTAGGTGTATGCTGTTCTGCT

GCCAACACTGGGCACAAGAGAGACACCAGGAACAAGAGAAACAAAAATTGCTGCCCTCCTGGTATTTATGCCCTATGGG

AGAATAGATGAAATACACAAGCAACGGAACAAGACAATGCCTGTGTATATCACCTGCGGGAACGGGCACCATCTTAGCT

GTAGGACTCTAGGGGGTCAGGTATGTGTACTTTCATGCAATCACCACCCAGACCAAGATAGGGAACTGTCAGGAATTCCC

ACCGTGGCAGAGCAGAAAAGAACCCAACTAGTATCCATGAGGATGTGGGTTCGATCCCTGGCCTCGCTCAGTGGGTTAAG

GATCCCGTGTTGCCATGAGCTGTGCTGTAGGTTGCAGACACGGCTCAGATCTGGCGTTGCTGTGGCTGTGGTGTAGGCCGG
CCGCTGCAGCTCCAAATTGACCCCTAACCTGGGAATCTCCATATGCCACAGGGGCAGCCCTTTAAAAAAAAAGCAAGATA
GAGAACTTTCCAGCACTGCTCCATCACTGGTCAGCTGACCTCTGACCTCTGTCGCCTTGGAGTCATCTTGCCTATTGGTCAT
GTGAAGGGAATGAGGTAGTCAGTACTCTTTTGTGACTACACCCTTCTGTGCATTTGTGTCTGTGGGAATCACTGTGGCTGTT
GCTGTGTGTTGCAATAGCCCTCCCATTGTCATTGTAGGGTGTGTATTGTATAGCCTTCCGTCATGTGACTAAGGGGTTTGTA
AGAACATATCACCGTTCATGGGTCCATTGTCACTCGCACTGAATCACATTTGAAACGTTGCTAGTTTGCGGCTTCTTAGAAT
AAAGCTTCTTTGAATATGACTGTCCCTGTCTTTTGGAATGCATGAGCATGAGCACACAGCCCTATTGGTATCTGACAAGGC
AGGGGTTCCAGGGGCAGAAGGCCTTACACACTACTGGTTTCCCAAAGGAGGTGTCCCACCAGCGGTGAATGATGGTTTAA
GGTACTCCTCGTAGTCAAGACCTGTTGTTGGTCTTTTACATTTGAGCCTATGTGGGGATCTCCTTGGATTAACGTTGCTCGTA
ACTTCTCCAAATCCGGTGATGTCAGGCACGCTTCCCTATGCTTCTTGACTTTTGGGAAAGGCCGTTCCAGACTTGGGCCTTT
TGATCATTGGGTGATAGGCTTGGCTATGCATTTTTGGAGATGTGTCCTTGGTAATTTGCAATAGATTTTCCCGGCTGAGCCTT
GTCTACATGCTGGACCCCCAAGCCAGCAACTCTATAGAGTAGCACACTTTGGGAACACCTGAGCTCCTAGAAACTTCCTCT
TAAGCAGACCGCTGGCCCACGTGCTTGGAAATTGCTTCCAGAGGATGCAGTGCAGAGGACGCATGGTGACTGGTCGATCA
GAGACAGGAAACATATACCTAGGATGTTCACGGAAGGGTGTGAAGTAGGAACCAACAGTCCCCAGATCCGTACATTGAC
TTAAGACTTGGATACCCCGCAATGGCAAAAGGAAGCAAAAAAAATATATATATAAGCATCTTCAGTTAAAGAAGAAAAA
AAAAAAGGCCATCTACCAGAGAGTCACGGAAGACGGGCCTCACCCTTCTCGGAACACCGCTGAATTCTAGAAGCTCTGA
CTCCCTCAGAGGTCTGGGAGCTTCATCCTAGAATGTATGCGCAGCCAAAATCCTAGTTCAGTGTCCACGAGCACGACATTT
CCACATATGCAAGGACTAACGCCACTTACTCGTGAGGAGCTCAGAAAATGCGGGCAGACAAGAAGGTCCCGAGTGGCAG
CTGTGCAACAGCCGGGACAGGGCGCTTTGGGGGCGTCTGAGGAAAGAGTGCAGACAGGATCCCAGACATTACAGAGGTG
CTGAAGCCACACGTCTGGAAGGACAAAGACTCCTGTCCCTGCCACAGGGGATTCCCAGCACTTCTGGCCTCACCCACAAA
GACACACGGCGCTACAGAACCATGAGCTCTTCACAACCCAGGACGAAGGAAGGAGAAACGGTGGCTGCGGAAGAGGCC
GCCCTTCTACAGAGCGTGCCCGGAGGATTCCTGCTCGAGGCGTCCCCAACAGGAACAAGAAATCTCAACACCCCTCAAGC
CCAGACAGCATCGCCAGGCACCCGCCTAAGACAGTGTCCCCACTCAGGCCCCAGGAAGCAGCAGGGCTGCTTGGAGAGC
TCAGGGGGGCCCGAAGGGAAAAAAAGATACCACACAGCAGGTGTGAGTGAGGCTTTACTGCTGATCAGGCAGGGCTGGG
TGGGGACCACACTGGGGGAGCAGGGCCTACATGCCGGGTCAGGGCTGGTCTTCAGGGCAGGGCTGGGCAGCTCAGGACT
GAGCCCCACAGGCAACGGCCAGGCTCCACACGTCAGGCCTGAGCTCTCCAGGTCAGGCTGCTCACAGCAGGAGCAAGCA
CAGAAAGGAAGGCGGGTCTGGTTGGCCTCAGAGCAGAAAGGATACCTTCAGCTGCTGACTTCACGGCAGAAGGGCGGGA
GGCTGCAGAGCGCTCAAGATCCTGGTGAATGCAGCCTGGGAAAGGCTTAGGGGCCACGTGAGGCCTGATGGGAACAAGG

AGGACAAGCAAGACGTCTGGTCGTGGGCTGCAGGGAGGGCCAGCTGTCCTCCAGGGCCTGGTCCTCACTCCATCTCCTGC

TTCCAGGGCACTACTTGTTGCCCCATGTGGCCACCTTTGTATACACGGGGTGTGGCAGGAAGCGGCTGGACCTCATGTAGG

CTGAGATCCTCTCCAGGCCCTCAAAGCGGCTGAGGAAGGCTTTCAGGTTTGGGAATTCATCCAGGCACTTGGGATCAAAT

ATGCGGTACAGGTCAAGGACATCGTAAGCCAGGAAATCCACATAGGTGAGCTGCAGAAAGGCAAAGCGTGCATGGGCAC

CAAACGCCGAACCTGCGAAAAATGACAACCCCGGGTCCCCAAAGCCGTCCCCTTACCTTGTCCCCTGCAAACCAAGGCCT

CTTCCCCAGAAACTCGGAGAAGGGCTTCATCTTTTCAGGGATCTCCTTCAAGTACCCAGGCTTCAGTTTCTCCTGAGGCAC

AAAACAGCTGTCACCACCCTCACACACAGACTCCCTGGCACACAGCAGGCCTCCCAGGGCTGGGCCGGATCCCAGCTGCC

AGCGAGCAGCCACGTCCCCCGACGGCACCTGTTTCGGTGCCCAGGCTATGCTTTAACCCACTGTGGTCATTAGAGTCCTAC

TGGGTCCCACCTCCACCTGTGAGAGGGGAACGGAAGGCAAAGGGCAAAGCCACCCTGTCCCTGAACCCGTCACCACTCA

GCTCCAAACACCTGCCATGGGTCAGACCAGCAGCGCTGTGAGACCTGGCAGAGCTCAGGCCTCAGCCCTCAGGCACATCA

ACACTCAAATGATTCGGGAAGGAAGAGCCTCTACGAAGCACTGCAAAGAGGGGGTAGAAATGCTGTGGATGCCTGAGCA

GTTTCTGGACAGTAGAGGAGAATCTGAGAGGCTGAAAGGAAGGAGGGAGAGGAACCAAGCTGGACCCTGTGCTGCCTAC

TGGACACAAGGGCTGACGCCTGAGATGCCCAGGAGGAGGCACTGGTCTGACGCAGTAAACACCCTGTCCAGGAAATGGG

AAAAAAAAAAAAAAAAAAAAAAGAAAAAAAAAAAAAAAA
